# Use of COVID-19 evidence in humanitarian settings: the need for dynamic guidance adapted to changing humanitarian crisis contexts

**DOI:** 10.1186/s13031-021-00418-w

**Published:** 2021-11-19

**Authors:** Alex Odlum, Rosemary James, Audrey Mahieu, Karl Blanchet, Chiara Altare, Neha Singh, Paul Spiegel

**Affiliations:** 1 Geneva Centre of Humanitarian Studies, Boulevard du Pont-d’Arve 28, 1205 Geneva, Switzerland; 2 Center for Humanitarian Health, Johns Hopkins Bloomberg School of Public Health, Baltimore, USA; 3 Health in Humanitarian Crises Centre, London School of Hygiene and Tropical Medicine, London, UK

**Keywords:** COVID-19, Humanitarian, Evidence, Guidance, Online platform

## Abstract

**Background:**

For humanitarian organisations to respond effectively to complex crises, they require access to up-to-date evidence-based guidance. The COVID-19 crisis has highlighted the importance of updating global guidance to context-specific and evolving needs in humanitarian settings. Our study aimed to understand the use of evidence-based guidance in humanitarian responses during COVID-19. Primary data collected during the rapidly evolving pandemic sheds new light on evidence-use processes in humanitarian response.

**Methods:**

We collected and analysed COVID-19 guidance documents, and conducted semi-structured interviews remotely with a variety of humanitarian organisations responding and adapting to the COVID-19 pandemic. We used the COVID-19 Humanitarian platform, a website established by three universities in March 2020, to solicit, collate and document these experiences and knowledge.

**Results:**

We analysed 131 guidance documents and conducted 80 interviews with humanitarian organisations, generating 61 published field experiences. Although COVID-19 guidance was quickly developed and disseminated in the initial phases of the crisis (from January to May 2020), updates or ongoing revision of the guidance has been limited. Interviews conducted between April and September 2020 showed that humanitarian organisations have responded to COVID-19 in innovative and context-specific ways, but have often had to adapt existing guidance to inform their operations in complex humanitarian settings.

**Conclusions:**

Experiences from the field indicate that humanitarian organisations consulted guidance to respond and adapt to COVID-19, but whether referring to available guidance indicates evidence use depends on its accessibility, coherence, contextual relevance and trustworthiness. Feedback loops through online platforms like the COVID-19 Humanitarian platform that relay details of these evidence-use processes to global guidance setters could improve future humanitarian response.

## Background

Humanitarian crises affect diverse peoples and contexts around the world, with an estimated 168 million people needing assistance and protection in 2020 [1]. For humanitarian organisations to respond effectively to complex crises, they require access to up-to-date evidence-based guidance detailing which interventions are recommended and which adaptations can be effective in certain contexts or circumstances. The need for evidence-informed humanitarian response is well-established [2–4]. More recently, however, the COVID-19 crisis has highlighted the importance of updating global evidence-based guidance to meet “context-specific and evolving needs in fragile settings” [5].

In this paper, we outline our review of humanitarian guidance documents and analyse the use of guidance in documented field experiences collected during the pandemic. By guidance, we mean normative documents and other media prescribing how to respond based on research evidence, including guidelines, recommendations, advice, standards, protocols and other related terms (Table 2). We uncover complexities in the way guidance has been used by humanitarian organisations during the COVID-19 pandemic, which deepens understandings of the process by which evidence is used in humanitarian practice. We argue that creating online feedback mechanisms and dynamically linking guidance with field experience could improve the use of evidence in current and future humanitarian response.

The origins of this paper can be traced to the COVID-19 Humanitarian platform (https://www.covid19humanitarian.com/), which the authors’ three universities built to facilitate access to guidance at the outset of the pandemic. Between March and August 2020, we reviewed 180 global level guidance documents and analysed 131. By December 2020, we had documented 61 field experiences shared by humanitarian organisations across three main domains: preparing for and responding to COVID-19, adapting existing interventions to COVID-19, and cross-cutting issues relevant to humanitarian settings. In addition, our team and partners facilitated weekly webinars on emerging and controversial issues attended by both public health experts and humanitarian practitioners working in crisis-affected countries (https://www.ready-initiative.org/webinars/).

Review and analysis of the guidance documents alongside data from field experiences collected through semi-structured interviews and posted to the COVID-19 Humanitarian platform (which can be accessed at https://www.covid19humanitarian.com/field_experiences) pointed towards two trends. First, although COVID-19 guidance documents were quickly developed and disseminated in the initial phases of the pandemic (from approximately January to May 2020), updates or ongoing revision of these guidance documents has been limited. Second, field experiences collected from April 2020 to September 2020 show that humanitarian organisations have responded to COVID-19 in innovative and context-specific ways, but have often had to adapt global guidance to inform their operations in complex humanitarian settings. We now situate these observations in the broader literature on evidence-based humanitarian response.

### Use of evidence in humanitarian response: what we know

Literature on evidenced-based humanitarian response has identified at least three factors that must be in place in order to achieve it: first, evidence must exist and be of sufficient quality [4]; second, it must be communicated to practitioners in understandable, useful and usable ways [6, 7]; and third, it must be used, implemented or otherwise applied by practitioners [8]. As Chynoweth et al. have stressed, not only is a strong evidence-base needed to improve humanitarian response, but also “it is important that humanitarian actors apply existing evidence” [8]. Similarly, Knox Clarke and Darcy’s review of the quality and use of evidence in humanitarian action made clear that the existence of quality evidence does not guarantee its use [9].

Researchers have identified a range of barriers that inhibit the use of evidence in humanitarian response, including unclear priorities, information gaps, the costs of addressing these gaps, and the dynamic nature of evolving crises [10]. At the same time, even if it is acknowledged that evidence is hard to come by, let alone to use, a trial and error mentality is also deemed ethically unacceptable given the humanitarian impacts at stake [11]. Combined, this reluctance to innovate but lack of evidence can lead to path dependency [10], or reliance on traditions, prior experience and intuition [12]. Experts have thus made a variety of suggestions on how to better incorporate evidence into humanitarian programming [13]. Among them is a suggestion that global evidence needs to be supplemented by local and context-specific knowledge [14].

Despite calls and recommendations to improve evidence-use in humanitarian response, examples of disconnect between evidence-based guidance and field realities persist. Tran and Hillen show how divides have emerged between the widely accepted guidance set out in Minimum Initial Service Package (MISP) for sexual and reproductive health (SRH), and contemporary realities of front-line humanitarian operations in this area [15]. In addition, Beek et al.’s review identified a lack of research on how the actual front-line practices of humanitarian responders map onto the objectives of multi-agency guidelines and agreements such as the MISP [16]. More recently, Khanpour et al.’s case study of WASH programmes in Uganda has shown that “effectively building decisions on the increasing amount of insights and information remains challenging”, and that various individual, organisational and environmental factors influence the use of evidence in the decision-making process [17]. More research into these complex, multi-factor processes by which guidance is used in humanitarian practice therefore seems necessary.

That is not to suggest literature on the general use of evidence in humanitarian policy and response, especially for decision-making, is absent. Rather, it is a growing field [9–12, 18–20]. However, among these studies, only a few empirically describe evidence-use processes in detail. For example, Darcy et al. document the use of evidence in Democratic Republic of the Congo (DRC), Ethiopia and Philippines, but “evidence” in these cases refers to assessment and monitoring information, rather than research-based or normative evidence [11]. Similarly, Knox Clarke and Darcy outline examples of “instrumental” use of evaluation evidence, but this is programme-specific rather than generalisable research evidence [9]. In addition, they highlight four examples where general research evidence has been used to shift global policies and paradigms: cash-based programming, minimum standards, cultural sensitivity, and early intervention [9]. But the intricate processes by which this normative evidence is then taken up in ground-level field operations remains, to our knowledge, understudied.

What these empirical studies of evidence-use have established is some consensus that humanitarian decision-makers use three main types and sources of evidence: pre-crisis contextual information; information concerning the nature of an evolving crisis; and (research or evaluation) evidence about what works [18]. Knox Clarke and Darcy reiterate this three-fold typology of early warning evidence, assessment and monitoring evidence, and evaluation evidence. In practice, however, these distinctions are not always maintained, and evidence-based response becomes described as a process in which all three types of information are used [9]. For example, Darcy et al. explain that evidence-based response is the process by which situational information is connected with established knowledge on the responses that work in such situations [11]. This has been described as response analysis; an analytical process by which response options are determined in a given situation [21]. At the same time, these authors have found that few humanitarian agencies actually conduct a formal or structured response analysis. So, if humanitarian actors do not use evidence in this way, how exactly do they use it?

Overall, while the need to adapt evidence to context is well established, few studies have unpacked what this translation of evidence to response looks like in practice. The complex processes by which operational organisations refer to evidence-based guidance when designing or adapting their response, particularly in complex and dynamic humanitarian settings such as those exacerbated by COVID-19, therefore remained an open question at the beginning of the pandemic.

### Evidence-use in a global pandemic: a new unknown

The ubiquitous impacts of COVID-19 across the world made it important to study evidence-use processes in humanitarian settings affected by the pandemic for multiple reasons. First, SARS-COV-2 was a novel virus and the scale of the pandemic was unprecedented, making many of its initial impacts on both health outcomes and societies unknown. This uncharted nature of the COVID-19 pandemic meant that compared to other humanitarian shocks, the existing evidence-base and tailored guidance for responding to COVID-19 was limited, at least early in the crisis until guidance was developed. Without a strong evidence-base but with urgent needs to respond, it was initially unclear how humanitarians would and indeed should go about programme design and adaptation.

Second, with travel curtailed and concerns about an infodemic as damaging as the epidemic itself, many humanitarians and academics alike - including the authors - felt compelled to help but constricted in how they could do so. The lack of primary evidence on COVID-19 opened an opportunity for information initiatives to compile and curate pre-existing wisdom from past epidemics, and to screen emerging evidence and guidance for quality and relevance. An example of this was Blanchet et al. who developed a list of 120 essential non-COVID-19 health interventions that needed to be maintained in poor countries based on existing model health benefit packages [22].

Third, many humanitarian actors felt bombarded with information from all angles, while traditional hierarchies of knowledge transfer were disrupted. In addition, breakdowns in relief supply chains coupled with an exodus of international staff in many organisations meant that local, bottom-up solutions to novel problems and field-level adaptations to context took on a new level of importance. There was thus an urgent need to capture details of these innovations, analyse the extent to which they were evidence-based, and disseminate their lessons to actors in other contexts looking for field-tested solutions and guidance.

Finally, the generation of evidence through robust evaluations could not be implemented quickly enough in a rapidly changing situation, especially as the priority was on response and even evaluation resources were focused on this responding. Ongoing monitoring systems were not immediately resilient to sweeping lockdowns and movement restrictions. This dearth of evaluation evidence meant humanitarian actors had to cobble together multiple sources of guidance and juxtapose these against field realities. How they went about this during an unprecedented pandemic provided an opportunity to study humanitarian evidence-use processes in action.

## Methodology

### Phases

**Fig. 1:**
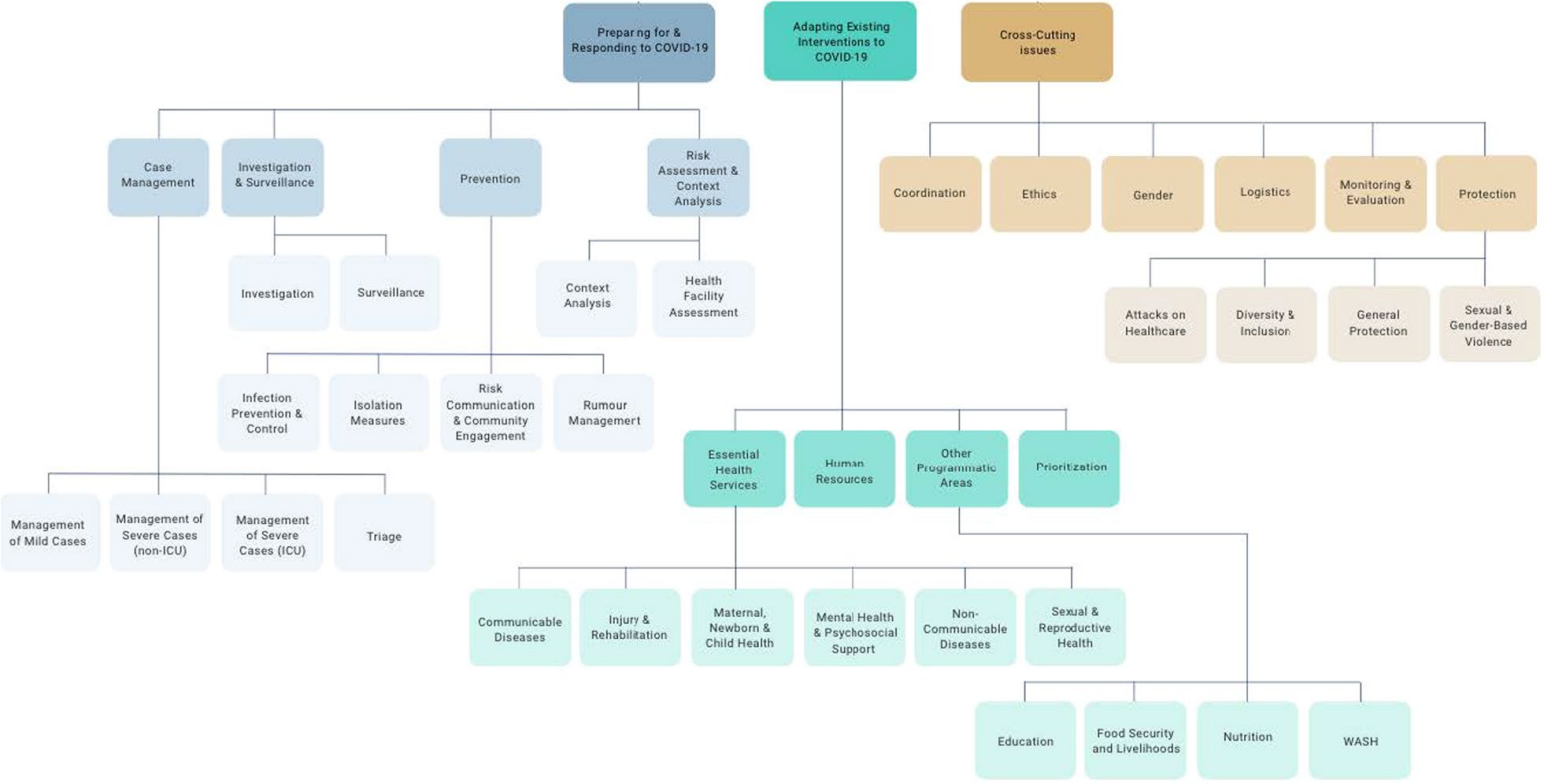
COVID-19 humanitarian platform–framework for guidance and field experience collection

Because of the dynamic nature of the pandemic and our intentions to provide timely and relevant information that immediately informed response, research for the COVID-19 Humanitarian platform evolved from a data collection and knowledge exchange exercise, into a research and analysis project. On reflection, this process can be summarised as involving four main phases. In the first phase (March 2020), we established research teams at the three universities, designed an analytical framework (Fig. 1), and set up the COVID-19 Humanitarian platform to collect and share guidance documents and field experiences for each section and area of the framework. The framework in Fig. 1 contains three main categories of interest: preparing and responding with COVID-19 specific interventions, adapting existing interventions to COVID-19, and cross-cutting issues. Each category contains multiple sub-sections, broken down into sub-areas where relevant. This framework served to structure the online platform and data collection process for both guidance and field experiences.

The second phase (April to September 2020) involved the definition of standard operating procedures to collect guidance and gather field experiences, collection of existing guidance documents on an ongoing basis (with no end-date cut-off), inductive learning from weekly webinars, and a series of qualitative interviews with humanitarian organisations on their responses and adaptations to COVID-19. We searched for guidance documents, assessed them following pre-established criteria, and posted relevant documents on the platform. In addition, we collected, reviewed and summarised field experiences into a standard format for the online platform. We actively collected field experience data from April until September 2020, with 54 out of 80 interviews conducted in May and June 2020. We continued interviews and posted some additional experiences until December 2020, but saturation, reduced capacity of the team, and the transition to analysis work slowed the rate of production. We learned that collecting experiences required active outreach and interviews, as only 12 people spontaneously submitted their experiences on the platform. The platform remains open to spontaneous submissions from humanitarian organisations, and continues to host the data for reference, but as of mid-2021 is mostly dormant.

Summary analysis of guidance documents and field experience data marked the third phase (August to September 2020). This analysis identified general trends about the nature of guidance documents and field experiences we had collected. Reflection on these trends generated a fourth phase of further analysis, which focused on studying the use of guidance by humanitarian organisations in the field and led to this paper.

### Collection and analysis of guidance documents

To identify available COVID-19 guidance documents, we actively prospected major humanitarian agencies’ websites and portals: United Nations (UN) agencies and the Inter-Agency Standing Committee (IASC), Centers for Disease Control and Prevention (CDC), The International Red Cross and Red Crescent Movement, global humanitarian clusters, international non-governmental organisations (INGOs), Reliefweb, and the Active Learning Network for Accountability and Performance in Humanitarian Action (ALNAP). In addition, we contacted humanitarian organisations to collect guidance documents on COVID-19. The guidance documents were individually assessed and we met on a weekly basis to discuss and validate those that met the criteria to be included on the COVID-19 Humanitarian Platform. Selection criteria required the guidance documents to be: evidence-based (based on research or operational learning); unbiased (e.g. not for promotional purposes); applicable to low income or humanitarian settings; and actionable. We then analysed the identified guidance documents using Microsoft Excel and Statistical Package for the Social Sciences (SPSS) to cross tabulate the number of documents per framework area, year and organisation type.

### Field experience interviews

#### Sampling

We solicited field experiences from humanitarian organisations via a form on the online COVID-19 Humanitarian platform, as well as through outreach across our research team’s own networks of practitioner contacts. Eligible interview participants were any employee of a humanitarian organisation that was responding or adapting interventions to COVID-19, who had sufficient operational knowledge of the intervention to describe it in detail. Participants based in country (national or sub-national levels) were preferred, but headquarter or regional level participants were also included if they had detailed knowledge of the context they were describing. This strategy led us to collect a range of experiences from UN agencies, major INGOs, government agencies, non-governmental organisations, and local civil society and grass-roots initiatives.

To mitigate selection bias, we tracked the geographic coverage of the field experiences collected, aiming to collect at least one experience from each of the humanitarian crises listed in United Nations Office for the Coordination of Humanitarian Affairs (UN OCHA’s) Global COVID-19 Response Plan [23], as well as the top five refugee hosting countries by number of refugees [24]. Similarly, we aimed to cover all sections and areas of the analytical framework in Fig. 1, to ensure data on a wide range of humanitarian interventions and techniques, and public health issues. The most frequently covered framework areas were risk communication, triage, context analysis, food security, particularly food, cash and voucher distributions, nutrition, infection prevention and control, mental health and psychosocial support (MHPSS), education, and sexual and gender-based violence (SGBV). Lebanon, Pakistan, South Sudan, Afghanistan, DRC, Bangladesh, Haiti, Libya, Nigeria, and Greece were the most frequently covered countries. A tabular breakdown of the interviews conducted per country and framework area can be viewed in our earlier commentary describing the COVID-19 Humanitarian platform: Singh et al. [25]

#### Data collection

The main data collection approach involved emailing contacts and representatives of humanitarian organisations to request their participation in a 30- to 60-min interview about their experience implementing or adapting programs to COVID-19. Interviews were conducted in English except one with a Haitian organisation which was conducted in French. Senior researchers at each university conducted the interviews or trained volunteer Master level students to conduct interviews alone or in conjunction with the senior researchers using a semi-structured topic guide. The guide contained prompts to assist enumerators to extract more detailed information from participants. Each interview aimed to focus on an area of the framework in Fig. 1 as specifically as possible: most organisations have responded to COVID-19 with multifaceted interventions, but we aimed to extract sufficient detail on a specific intervention by focusing on one key area at the lowest possible level of the framework. After conducting the interviews, we cleaned notes and transcripts and drafted summaries according to standardised templates that covered context, intervention and rationale for adaptation, process modalities, challenges and enabling factors, and related documents.

Each field experience summary underwent a two-stage review process: firstly, a panel including the authors and other senior researchers from each of the three universities involved in the COVID-19 Humanitarian project reviewed the field experience summary; secondly, we sent the finalised summary to the concerned organisation for review and final approval to publish. We consulted the “Consolidated criteria for reporting qualitative research checklist (COREQ)” to reflect on our methods and identify limitations, to consider when analysing the data and drawing conclusions [26].

#### Data analysis

We conducted structured qualitative content analysis of the field experience interviews using NVivo Version 12 Pro software. We considered the final published field experience as the main data source, as these were clean and standardised allowing for the most comparable interpretations across experiences. Where clarity or further detail was needed, we referred back to the interview recordings, transcripts or notes. After initial familiarisation of the data, the first and second authors coded the field experience summaries to analyse specific questions about the usage and usefulness of COVID-19 guidance by humanitarian organisations. We specifically searched for three types of guidance use in our coding exercise: whether the organisation referred to guidance explicitly or implicitly; whether the organisation used the guidance directly, indirectly, or not at all; and whether the organisation evaluated the guidance positively, negatively or missing. At a second stage, we checked for further references to guidance by querying all text for a long list of synonyms and words related to “guidance”, including: guidelines, recommendations, advice, standards, and protocols (see Table 2).

## Results

### Guidance documents

**Fig. 2:**
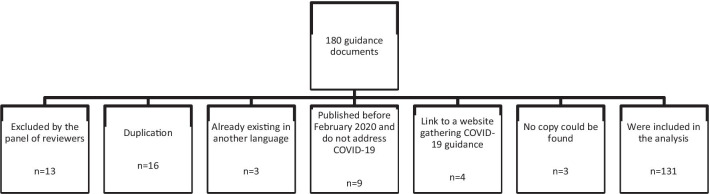
Selection of guidance documents for analysis

In total, we identified and reviewed 180 guidance documents. This analysis considers 131 documents, with 49 guidance documents excluded from the analysis for the following reasons stated in Fig. 2.

Out of 180 guidance documents identified and reviewed, our weekly review panel discarded 13 because they did not meet the selection criteria: they either were not evidence-based, not actionable, biased, or did not apply to either low-income or humanitarian settings. Sixteen guidance documents were duplications (the same document found by different researchers) and three were already included in the dataset in another language. Nine guidance documents were published before 2020 (some were published in 2017 or 2019) and did not specifically address COVID-19. They were either guidance documents for past epidemics or generic guidance documents on risk communication and community engagement (RCCE). Four were not guidance documents but links to other guidance gathering websites. The copies of three guidance documents listed in the dataset could not be found at the analysis stage neither in the web-based platform, nor in the web by typing their title in the search engine.

Ultimately, the guidance documents covered a wide-range of the COVID-19 framework areas depicted in Fig. 1. We found guidance for all but five areas at the lowest level of the framework. Often a single guidance document covered multiple areas, so we were satisfied that the whole framework was covered with at least one document.

**Fig. 3:**
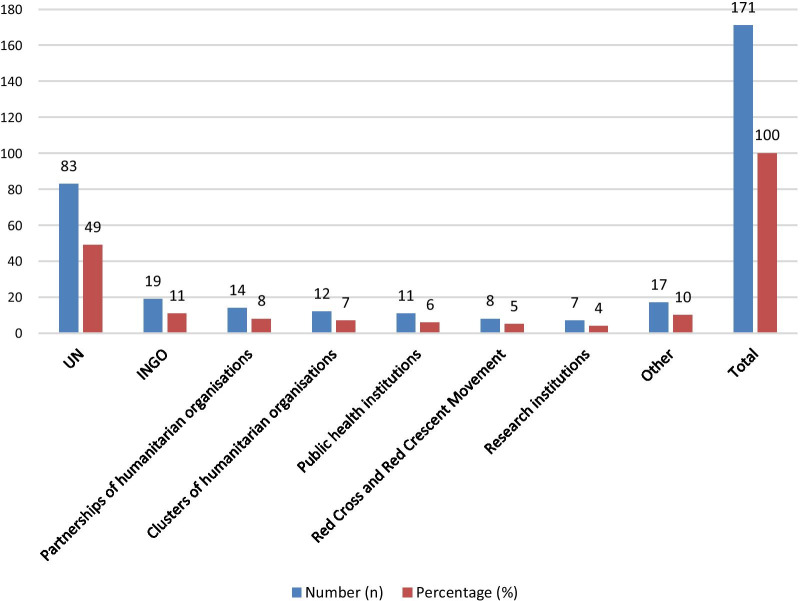
Number and percentage of guidance documents (co-)published per type of organisation

Figure 3 shows the number of guidance documents published or co-published per type of organisation. In total, 64 unique organisations published 131 documents. Some organisations published multiple documents, and some co-publications involved multiple organisation types. This resulted in 171 publishing organisations. Out of the 131 guidance documents included for analysis, 31 (24 %) were published or co-published by World Health Organization (WHO), 20 (15 %) by United Nations Children’s Fund (UNICEF), 10 (8 %) by IASC, 9 (7 %) by United Nations High Commissioner for Refugees, 6 (5 %) by World Food Programme, and 6 (5 %) by United Nations Population Fund. A wide range of organisations produced five or fewer of the guidance documents.

In terms of relevance, out of the 131 guidance documents, 55 (42.1 %) were relevant to all settings, 39 (30 %) were relevant to low and middle-income countries, two (1.5 %) were relevant to low-income countries and 35 (27 %) were relevant to humanitarian settings. All 131 guidance documents included were available in English. Only 37 (28 %) also existed in French and 19 (14 %) in Arabic, despite these being common languages in many humanitarian settings.

We identified relevant guidance documents published between 30 January 2020 and 31 May of 2020, 79 % of which originated in March and end of April 2020. As of August 2020, only 24 (18.3 %) of the 131 guidance documents had been updated since their first publication, despite the swiftly evolving knowledge and evidence-base on the nature of SARS-COV-2 and its impacts, as well as continually evolving lessons on effective public health and humanitarian response modalities to the COVID-19 pandemic. Further cross tabulation analysis showed that the WHO and the CDC were among the first organisations to publish guidance documents, while guidance documents from the Africa CDC were not published until later in April 2020.

### Field experiences

Between April and December 2020, the team conducted 80 semi-structured interviews with humanitarian workers from local civil society and grassroots initiatives, international and national NGOs and UN agencies based primarily in the field. This process shown in Table 1 generated 61 field experience summaries spanning 40 countries that underwent a standardised review by the three universities before being uploaded to the COVID-19 Humanitarian platform. Bold rows in Table 1 represent the sum of the non-bold numbers in the rows above.

**Table 1:** Number of submissions, interviews and published field experience summaries on to COVID-19 Humanitarian platform per status

Data collection process	Number (n)
Total online submissions leading to interview	12
Researcher outreach leading to interview	68
**Total interviews completed**	**80**
Summary drafting process incomplete/dropped	(-8)
Summary excluded after interview (insufficient information)	(-8)
Summary excluded after drafting (panel rejected)	(-3)
**Total field experience summaries published**	**61**

In total, 14 organisations submitted their experiences via the online form, but two were spam and immediately rejected. The 12 genuine submissions nonetheless required curation and a follow up interview to convert submissions into the standard required for published field experiences. The other 68 interviews were conducted with the contacts of our research team and snowballed participants, via phone or video calls.

After conducting the interviews, we excluded eight cases for lacking sufficient data, because the initial information collected was too general or incomplete, and pursuing further details would not have been feasible or too costly. For example, if after prodding or follow-up, the interviewee revealed no more specific or detailed information than what could be found on the organisation’s website or other published communication materials about their activities, we dropped the process. Panel reviewers also found three interventions to be irrelevant, controversial, or unsuitable for further promotion, even in light of their specific context. We decided to exclude these data after deliberation and based on consensus. Eight interviews could not be converted to published summaries due to researcher unavailability or remained in process at the time of analysis and writing. Singh et al. [25] summarised in more detail the field experiences collected for the COVID-19 Humanitarian platform.

### References to guidance in the field experiences

The field experience interviews focused on soliciting detailed descriptions of the adaptations and interventions humanitarian organisations had made or were making to continue operating in the context of the COVID-19 pandemic. Analysing the summarised field experiences, we found multiple instances where organisations explicitly mentioned guidance (such as “WHO guidelines for personal protective equipment”) when describing their interventions and adaptions to COVID-19 (https://www.covid19humanitarian.com/field_experience/?id=110). In addition, we found cases where humanitarian organisations made implicit references to guidance, for example when a participant explained the way staff followed or departed from “recommended” procedures (https://www.covid19humanitarian.com/field_experience/?id=134). In these references to guidance, we identified three major themes: organisations refer to authoritative global or international guidance and adapt it to context; organisations refer to guidance disseminated from headquarter levels and adapt it to context; organisations combine various sources of guidance and adapt it to context. While our sampling strategy did not allow us to draw conclusions about which approach is more frequent among humanitarian organisations, the following examples demonstrate the variety of ways guidance is used and adapted.

#### Adoption and adaptation of global guidance

Humanitarian organisations that explicitly mentioned guidance when explaining their COVID-19 responses and adaptations often referred to documents issued by WHO and the IASC, two predominant global bodies for public health and international humanitarian response respectively. In particular, respondents referenced WHO guidance documents on a variety of topics, including case management, personal protective equipment (PPE), case definitions, RCCE, infection prevention and control (IPC), screening, and psychosocial support (PSS). For example, War Child’s psychosocial support interventions were adapted based on the Lebanon’s Ministry of Health (MoH), WHO, and IASC guidelines and from the local coordination group headed by UNICEF (https://www.covid19humanitarian.com/field_experience/?id=48). Organisations also specifically mentioned global guidance developed by academic institutions, such as the London School of Hygiene and Tropical Medicine (LSHTM) guidance for preventing COVID-19 infections among high-risk individual in camps and camp-like settings [27] (https://www.covid19humanitarian.com/field_experience/?id=117).

In addition to citing guidelines and documents, humanitarian organisations explicitly referred to guidance in the form of other communications materials issued by authoritative humanitarian and public health agencies like WHO and IASC. They also explicitly referred to materials issued by local and national governments, such as lists of frequently asked questions and answers (FAQs), posters or leaflets, and social media material with key messages. An example of this was explained by the Mixed Migration Centre, who drew on FAQs from WHO to verbally share information on COVID-19 with respondents after conducting phone interviews to collect data on migration trends (https://www.covid19humanitarian.com/field_experience/?id=65).

References to guidance were not limited to documents and key message materials, but also covered other types of global standards and resources, including methodologies, models or indices. For example, in DRC, REACH worked with UN OCHA and a core group of information management actors to rapidly develop an index for measuring the level of vulnerability of each health zone during COVID-19, and the likelihood of negative impacts of the epidemic at this level (https://www.covid19humanitarian.com/field_experience/?id=57). To do so, they referred to existing global vulnerability models, namely the European Commission’s Index for Risk Management (INFORM) severity index [28], but adapted it substantially to the local context.

Common across these references to international or global guidance was an additional explanation of how guidance was adapted, amended, translated, or otherwise contextualised. For example, an anonymous organisation in Iraq who described their health facility triage system explained how their volunteer distributed leaflets about COVID-19 were designed based on WHO guidelines, and translated by the organisation into Arabic (https://www.covid19humanitarian.com/field_experience/?id=110). In this same case, the organisation also explained how they had referred to WHO guidelines for PPE, but had to ration usage of PPE because supply and stock lines were limited, and thus could not fully align their implementation with the guidelines.

#### Dissemination of headquarter level guidance

In multiple cases, organisations at national and local levels adapted or generated guidelines with assistance from their global headquarters. The International Rescue Committee’s (IRC) shift to remote delivery of essential health services during a strict urban lockdown in Jordan involved close collaboration between the country office and headquarters (https://www.covid19humanitarian.com/field_experience/?id=105). Firstly, technical documents from the IRC headquarters aided country intervention teams to develop their remote service delivery plans for primary healthcare and reproductive health clinics. Secondly, experts well-versed in this content immediately trained the teams on these new protocols via a webinar. As such, the rapid design of protocols and staff training allowed for an adaptation of services with only one week of interruption.

In a similar case, the Libyan Red Crescent Society (LRCS) received support from the International Federation of Red Cross and Red Crescent Societies (IFRC) to develop an appropriate COVID-19 RCCE strategy and virtual training for volunteers due to travel limitations (https://www.covid19humanitarian.com/field_experience/?id=112). Another example of top-down dissemination from headquarters was the COVID-19 preparedness and response activities in Médecins Sans Frontières’ (MSF) field hospital in Cox’s Bazaar, Bangladesh (https://www.covid19humanitarian.com/field_experience/?id=102). In this case, the field hospital used guidelines on disinfection and sterilisation, as well as on prone position (for the management of severe cases), which had been generated by MSF headquarters based on WHO guidelines. In a similar way, IRC’s MHPSS programming in Tanzania utilised IRC’s global COVID-19 MHPSS operational guidance [29] to define key areas for adaptation (https://www.covid19humanitarian.com/field_experience/?id=93). A similar strategy did not directly stem from headquarter guidance on COVID-19, but drew on past organisational experience to inform current response and adaptation. For example, MSF in Central African Republic (CAR) quickly adapted their existing PPE guidelines based on previous experience from Ebola and Cholera outbreaks and in consultation with IPC experts (https://www.covid19humanitarian.com/field_experience/?id=18).

#### Combination of multiple guidance sources

Humanitarian organisations also combined multiple sources of guidance to create procedures fit for the context in which they were operating, including language, culture, and resource-specific considerations. For instance, in describing their work on sensitising communities on COVID-19 and advocating for the protection of women from SGBV during the pandemic, Action Aid Haiti explained how they referred to guidelines from WHO, the Haitian MoH and Action Aid International, but adapted these documents to the local context (https://www.covid19humanitarian.com/field_experience/?id=53). Similarly, in South Sudan, Islamic Relief mentioned that it had been useful to adapt guidelines from WHO, national and local authorities, to the local context to inform their health surveillance operations in porous border areas near Kenya (https://www.covid19humanitarian.com/field_experience/?id=55). Doctors with Africa (CUAMM)’s operations in South Sudan also described needing to adapt their organisation’s general guidelines for triage into a simplified version suitable for the specific context of a local health facility (https://www.covid19humanitarian.com/field_experience/?id=75).

A variation on the approach of combining global with national and local guidelines was to refer to domestic guidelines designed for a foreign context in the context at hand. Medical Volunteers International, an INGO which operates health clinics in Greece, filled specific clinical gaps with foreign guidance issued from the UK’s National Institute for Health and Care Excellence (NICE) (https://www.covid19humanitarian.com/field_experience/?id=52). The organisation deemed the NICE infection prevention and control guidelines more appropriate than material issued by the Hellenic National Public Health Organisation, which although context-specific to Greece, targeted the public rather than the specialised health clinic settings where they operated and for which they needed guidance.

### Additional references to guidance

During our analysis, we recognised that the word “guidance”, or related stemmed words such as guide and guideline, did not cover the full spectrum of ways in which humanitarian organisations refer to their use of evidence when explaining their experiences responding and adapting to COVID-19. To address potential gaps in our analysis, we ran a query on the published field experience texts to identify synonyms and stemmed words that would also indicate reference to and usage of guidance by humanitarian organisations. This allowed us to gain more and deeper insights on the extent to which humanitarian responses and adaptations to COVID-19 were evidence-based, as well as the nature of evidence and guidance usage in programme design.

We first identified a long list of 40 synonyms for guidance and guidelines, looking for relevant terms in publicly available web-based dictionaries and thesauri (e.g., www.thesaurus.com). After running the long query list, we dropped 19 terms that returned no relevant matches or where the usage of the term did not imply reliance on, following or use of guidance. For example, if the example showed the organisation *provided* “guidance” to beneficiaries on handwashing, we excluded it. If, however, it implied the organisation *followed* guidance (or its synonyms) on handwashing, we included it. In total we identified 85 examples for 21 terms.

Table 2 presents a selected example for each search term. While these are not directly transcribed quotes from organisations (they are extracted from the editorialised and published field experience summaries), the examples indicate the breadth of ways organisations refer to guidance, and suggest complexities in the way humanitarian organisations use guidance, and by extension, base their responses on evidence.

**Table 2:** Examples of guidance-related terms identified in the published field experience summaries

Search term(s)	Number of exact matches	Number with stemmed words	Number of examples identified	Selected example of how the search term was referred to in the published field experience summaries
Guidance	26	26	13	The global nutrition community was still developing and issuing guidance on anthropometric measurements
Standard (Standards)	7	14	9	There is a gap of 52% or 2099 beds in total the number of available hospital beds compared to global standards
Protocol	20	40	8	The survey team adjusted their anthropometric- measurement protocol according to international recommendations that caution against height and weight measurements involving prolonged contact
Guideline	2	11	6	Each session follows guidelines from CDP’s pre-existing MHPSS services that have been adapted to COVID-19 and the online format in consultation with specialised psychologists
Rules (Rule)	12	15	5	For example, staff and communities still tend to interact in breach of physical distancing rules
Procedure (Procedures)	5	19	5	The COVID-19 standard operating procedure protocol circulated by the national government, which was adapted from the WHO, was used to develop this training curriculum
Recommendation (Recommendation, Recommended)	4	12	5	International Medical Corps did not have a large stock of Mid-Upper Arm Circumference tapes prepositioned to meet the recommendation of using one tape per household
Measures (Measure, Measurement)	98	119	4	All hospital staff underwent a one-week training on IPC measures in hospitals and communities
Plan (Plans, Planned)	39	67	4	Shortly after, the UN Global Humanitarian Response Plan was enacted in CAR. The response plan included awareness-raising sessions, creation of isolation spaces, reinforcement of handwashing and strengthening of epidemiologic surveillance, although these were slow to be implemented
Principle (Principles)	3	5	4	The “no touch” principle was encouraged, especially during small group meetings or home visits
Requirement (Requirements, Required)	1	52	4	Reusable PPE was chosen for three reasons: (1) for reasons of space as reusable PPE takes up less space than the required stock of disposable PPE
Instruction (Instructed, Instructions)	0	12	4	Acceptance of instructions to protect themselves appears to have increased, especially after the government began reporting increases in numbers of cases
Guide (Guided, Guiding)	4	39	3	IRC Jordan developed a remote delivery plan guided by technical documents from IRC headquarters
Process (Processes)	31	42	2	At this time, our usual process for inspection of samples and quality checks using certifications, testing, and other processes has been modified to support a distributed workforce, and an evolving list of approved products by the Food and Drug Administration
Advice	10	10	2	Limited coordination between headquarter, regional and local offices at the beginning of the response, which led to inconsistency in the tools, advice and instructions shared
Evidence	8	8	2	This program features a community-based model and utilises evidence-based approaches to group interpersonal therapy for the refugee community
Information	93	133	1	At a time when emergency use authorisations are implemented and information about COVID-19 changes regularly, Americares has dedicated more manpower to keep pace with the changing standards
Form	16	23	1	For half a day, a team of two trained MSF staff, one IPC/logistician and one nurse, evaluate the health structure using a specific assessment form which focuses on IPC, the flow of patients and health workers
Criteria (Criterion)	3	3	1	Beneficiary triage into risk groups is carried out in alignment with the guidelines and criteria that were approved by the SGBV working group in Lebanon
Directive (Directives)	1	39	1	Following the MoH’s directive for contingency health promotion on COVID-19…
Regulation (Regulations)	1	6	1	ACF adapted their activities to tackle constraints related to the COVID-19 situation, and to best support and protect staff and beneficiaries, following government and Inter Sector Coordination Group (ISCG) regulation
Total	384	695	85	21

## Discussion

The literature on evidence-based humanitarian response argues it should take into account data and information derived from reliable assessments of needs, and from evaluations of intervention effectiveness [16, 30]. Given the complexities of conducting research in humanitarian settings, it is not expected that a perfect evidence-base will exist [31]. Instead, a well-informed response decision is one that “takes due account of data and information most relevant to the crisis context, and combines this with experience-based knowledge to determine what intervention is the most appropriate in that context” [11]. Despite this pragmatism, it is often lamented that many humanitarian interventions remain insufficiently based in evidence [30, 32–34]. A core assumption of these studies, however, is that we know what the process of using evidence looks like in detail. In this discussion, we show how our data depicting the ways guidance is referred to in humanitarian field experiences help paint a richer picture on the nature of evidence-use in humanitarian practice, in the context of the fast-moving COVID-19 pandemic.

### Key factors for using evidence-based guidance in humanitarian response

Our analysis of humanitarian organisations’ field experiences when responding and adapting to COVID-19 identified four factors that may indicate whether the use of guidance indicates an evidence-based humanitarian response: the availability of guidance and access to it; coherence and coordination between multiple guidance sources; contextual relevance of the guidance; and trust and credibility of the guidance. We argue these factors can indicate whether humanitarian response is, or can be said to be, based in evidence, particularly in dynamic and uncertain humanitarian settings affected by the COVID-19 pandemic.

#### Availability and access

Within a few months of the pandemic, guidance documents were available on a wide range of framework areas, and our interviews only identified a few organisations that mentioned specific gaps in available guidance. For example, one organisation lacked guidance for programming with mobile populations that was specific enough to the complexities of migration journeys in COVID-19 affected settings (https://www.covid19humanitarian.com/field_experience/?id=94). Another found that guidance on the modalities and timing for resuming activities and transport post-lockdown was missing (https://www.covid19humanitarian.com/field_experience/?id=52). Most participants, however, did not suggest guidance was unavailable, and some even complained of information overload.

Initially, our guidance document review found some framework areas to be under-documented, such as sexual and reproductive health, human resources and coordination. However, on closer analysis, these topics were often included within documents categorised under other framework areas. For instance, guidance documents on adapting existing food security and livelihoods interventions to COVID-19 often include a section on the safety of staff during food distribution activities, and a section on how to coordinate with other stakeholders. Guidance documents on maternal, newborn and child health often include a section on sexual and reproductive health. Thus, guidance on most framework areas was available, even if some topics were more difficult to access by being bound up among broader topics.

Clearly, availability of evidence-based guidance is a key first step in order for humanitarian organisations to base their implementations on it [4]. In the case of COVID-19 in humanitarian settings, it seems that guidance was available, particularly from April onwards when many documents became available. Alone, however, the availability of guidance is insufficient to say it has been used: access is also crucial. Here we identified some problems. Although we found guidance documents were available from a wide range of organisations (Fig. 3), we identified some limitations in the way large INGOs disseminate guidance documents. During the initial review period (March to May 2020), we could not identify guidance documents from major INGOs including MSF, Save the Children, and BRAC, as they did not publish them openly on their organisational websites nor on general humanitarian resource websites (such as Reliefweb or humanitarian cluster websites). We later collected guidance documents from those organisations directly after interviews with their staff or following a more detailed investigation and personal follow-up. We learned that although these organisations had developed various COVID-19 guidance documents, they had initially kept these documents internal.

This situation has two implications for our understanding of evidence-use processes. First, even if evidence is not accessible to the broader public, it may still be privately accessible, and therefore could still have been used for response. Second, conversely, if evidence is kept private, smaller organisations may lack the means to access it, and thus not have used it to inform their response.

#### Coherence and coordination

While making evidence-based guidance available and accessible can help organisations to use it, striking a coherent and coordinated balance of quantity and quality, breadth and specificity, and the format of dissemination, over time, is also important to ensure that using guidance equates to basing programmes on suitable, up-to-date evidence. On the one hand, our interviews revealed cases where the guidance available was too general or designed for a broad public audience, rather than tailored to the health and humanitarian professionals who actually implement humanitarian programmes. On the other hand, some organisations found FAQs, information sheets, key messages and other communications materials (as opposed to detailed technical guidance documents) helpful when creating localised response plans and procedures. In addition, our interviews showed many cases of organisations collating global guidance documents with local, national, regional materials, as well as mixing guidance of various formats together. Such practices illustrate the importance of disseminating guidance in a wide range of formats available to ensure it is widely used.

However, disseminating evidence in a variety of formats also places an onus on guidance setters to ensure coherence between these different types of documents and formats. Multiple studies have called for more appropriate guidance on COVID-19 [35, 36]. Our interview respondents also mentioned challenging discrepancies between different COVID-19 guidance documents, for example, between those disseminated by global (UN) organisations, other humanitarian organisations, and national (MoH) institutions. This variation made it difficult for humanitarian organisations to decide which guidance to follow. In Central African Republic (CAR), MSF described efforts to align guidance with local partner organisations as a fragmented, decentralised process (https://www.covid19humanitarian.com/field_experience/?id=18). It follows that if guidance is incoherent, simply relying on either a single guidance source or an idiosyncratic combination of guidance does not indicate that an intervention is based on evidence. In addition, comparative analysis of sources is also needed to justify the connection between the evidence and guidance in the context.

Two other factors are also relevant to ensuring coherence. The first is timeliness. The evolving evidence base on COVID-19 meant that relevance of guidance was also rapidly changing. If organisations used guidance, it does not necessarily mean they have engaged in an evidence-based response, as the guidance used may have been outdated. The second factor is information coordination. The flurry of information service efforts that emerged early in the pandemic, while well intentioned, also generated duplication and confusion. Our own COVID-19 Humanitarian platform project experienced this, when we found an individual website had created similar online compilation of guidance documents, prompting us to reach out and coordinate efforts. The need for improved information management and coordination, at all levels of humanitarian response, has long been recognised [37]. COVID-19 has only reiterated the need for coordination to ensure guidance disseminated online is updated, aligned, contextually grounded and responsive to the evolving situation.

#### Contextual relevance

While using coherent and up-to-date guidance can help responses to be grounded in evidence, it is important for guidance to also be appropriate and sensitive to context, both in terms of language and technical realities. Adapting guidance to context was widespread among humanitarian organisations responding to COVID-19 in our sample. Such adaptation is a natural and often encouraged [14], but also raises an important question: at what point does the adaptation of guidance to context lead it too far away from the original parameters of the evidence on which it is based?

In our interviews, multiple organisations explained how global guidance documents needed to be translated and adapted to their local language, which took time and often required expert consultation. The need for translation applied not only to new COVID-19 specific documents, but also to evidence reviews from past epidemics such as cholera and Ebola, which remain published mainly in English. Literature on translation in crises is emerging [38–41], and some good practices for translation certainly exist; the Africa CDC systematically translates its guidance documents into French, Arabic, and Portuguese, for instance. Literature is also emerging on how to enhance translation for humanitarian settings. However, even with major languages covered, translation to the hundreds of languages used at the operational level would require more innovative solutions, including capitalising on automated translation and transcription technologies and natural language processing tools. In both cases, traditional or automated translation adds a risk that the original evidence-base is not precisely conveyed in the translated guidance. Many times, this may be a matter of mere nuance or precision. Nonetheless, it suggests a gap between the use of translated guidance and the use of evidence that needs further investigation.

Similar to translation processes, adapting indicators to context adds complexity to the use of evidence-based guidance. REACH encountered multiple challenges to design a severity index that was sensitive to local complexities and flexible enough to handle dynamic and uncertain data generation (https://www.covid19humanitarian.com/field_experience/?id=57). For instance, they had to refine the general list of indicators for severity to a more limited set when they examined the (lack of) locally available data at the level of the health zone, weight for differences in the quality of data across indicators and zones, and take into consideration the secondary impacts of COVID-19, such as on food markets and employment. While these context-specific amendments likely improve the relevance of the model in country, it also widens the gap between the local version of the index, and the evidence-base underpinning the global model.

#### Trust and credibility

Finally, in many cases, trust was crucial for effectively implementing guidance. But building trust has been complicated in COVID-19 responses [42], whether with governments, local authorities, or affected communities, due to widespread rumours, misinformation, competing narratives and alternative beliefs that run counter to evidence-based guidance. We encountered multiple examples where humanitarian organisations had to tread carefully to build trust before launching their response. In refugee camps on Lesbos, Greece, MSF described that guidance to isolate vulnerable persons from the broader camp population was not an approach supported by local authorities, hence alternative approaches had to be discussed before evidence-based programmes could be implemented (https://www.covid19humanitarian.com/field_experience/?id=63). In other contexts, communication with local authorities including the police force, as well as collaboration with MoH, UN organisations, other INGOs and NGOs, were also integral for organisations to build space and confidence to introduce new or adapt existing interventions. Engagement with the national MoH was particularly important for a wide range of tasks: gaining permission to access sensitive areas, using national hotlines, surveillance and reporting, securing additional supplies, receiving weekly updates, building strategies and action plans, or deploying staff to/from the government response. At times, organisations reporting good relationships with the local health authorities also experienced success in gaining trust of the local population, though this may be contingent on the community’s own relationship with authorities.

In general, whether trust of the community, government, or both were required in a given context, successful implementation of guidance was heavily dependent on securing the requisite trust, which can mean at least acknowledging ideas that run counter to the evidence-base. We do not know how these trust-building processes were undertaken in detail in each case. However, if in order to use evidence, organisations need to at least consult with actors who follow guidance that is *not* evidence-based, it adds important complexity to the process of evidence-use as a whole.

### Implications for evidence-based humanitarian response

Guidance, when considered broadly, played an important role in the design and implementation of responses and adaptations to COVID-19. The wide range of terms organisations used to refer to guidance (Table 2) show that they are concerned with applying various evidence-based standards and protocols, or at least comparing their responses with existing benchmarks and documents. This suggests that evidence-based humanitarian response may be more prevalent than previously expected. At the same time, establishing whether using available guidance is a solid indicator of using evidence, requires consideration of processes related to accessibility, coherence, contextual relevance, and trustworthiness.

Thus, to understand evidence-use, it is important to collected detailed process level information on how it is being used, and relay this information to guidance setters in a more dynamic feedback loop. The COVID-19 Humanitarian platform provides a potential model for a more circular, dynamic and responsive evidence-based guidance development and implementation process. By combining guidance documents with detailed experiences and anecdotes from the field, documented via qualitative case studies, surveys, webinars, and discussion boards, the platform offers users the chance to consult what is recommended based on evidence, alongside what is actually happening on the ground. Elsewhere in the literature, feedback mechanisms, knowledge sharing, and research-practice partnerships are also being explored in creative ways [43–45].

Such a feedback loop could complement the prevailing guidance development model, which relies on updating existing guidance from previous epidemics, and which may not necessarily be relevant to the specific contexts and challenges of contemporary crises. It may also help to counter information asymmetries, such as those experienced in the early phases of COVID-19: transmission patterns from East to West/North before reaching the South led to a proliferation guidance designed for advanced economies, with limited relevance for low-income and humanitarian crisis settings. Incorporating documented qualitative experiences from these settings could therefore help to generate genuinely global guidance.

The COVID-19 Humanitarian Platform certainly has room to improve, especially in terms of attracting and sustaining users, funding, and spontaneous submissions (it only received 12), increasing the speed of knowledge exchange, as well as presenting and communicating information in a more engaging manner. Nonetheless, with refinement, it could serve as a basis for more dynamic and responsive evidence implementation that connects field-level experiences with global and headquarter level guidance setters.

### Limitations

To our knowledge, this is the first study investigating the use of COVID-19 guidance in humanitarian settings. It covers a wide range of humanitarian settings and organisations, and the in-depth interviews reveal details on multiple aspects of response and adaptation to COVID-19, including processes of guidance and evidence use. However, few interviews focused explicitly or solely on the use of guidance. Rather, in explaining the detailed modalities and rationales behind their interventions and adaptations, organisations made natural references to various global and national level guidance sources, as well as internal guidelines, protocols or procedures. A strength of this approach is that we avoided priming respondents to mention guidance which would otherwise have been omitted. A weakness is that we may have missed further complexities in the ways which organisations used and referred to guidance.

Another limitation of the study design is the risk of selection bias among the organisations interviewed. Although we attempted to ensure diversity of interview participants by tracking location and type of organisation, humanitarian organisations may have self-selected into the study, for example by completing the online form to promote their interventions or because they had sufficient time or resources to complete the form. In addition, the use of a convenience approach to sampling and snowballing participants from our own connections meant the coverage of contexts likely favours locations and contexts where the research team had previously worked or conducted research, and may omit other important humanitarian contexts. Nonetheless, we remain confident our sample reflects an informatively diverse range of organisations and humanitarian contexts for our qualitative approach.

## Conclusions

This study aimed to analyse the use of global humanitarian guidance by humanitarian actors according to their field experiences of COVID-19 responses and adaptations. Experiences from the various crisis settings indicated that adopting guidance to respond and adapt to COVID-19 is not a linear process, but rather a complex interplay involving substantial innovation and adaptation, to meet the demands and constraints of the local context. We found that organisations refer to guidance and evidence-based processes with myriad terms, and use guidance in three main ways: adopting global guidance, using guidance disseminated from within their organisation, or combining multiple sources of guidance. However, in most cases, organisations had to adapt this guidance to context. This adaptive process involved four key factors that reveal details about the use of guidance in humanitarian response: access and availability, coherence and coordination, contextual relevance, and trust and credibility. However, unless we know more about the relationships between guidance and field experience as they evolve, it is hard to assess the extent to which responses that use guidance are truly based on the best and latest evidence.

Our findings imply that the development of guidance aimed at humanitarian actors could be enhanced through responsive incorporation of contextualised field experiences in a timely manner, using feedback loops that link global evidence-based guidance with ground-level realities. The COVID-19 Humanitarian platform offers a model for this more nuanced connection of evidence and experience-based response, and sows the seeds for further process level research on evidence-use in humanitarian settings.

## Data Availability

The COVID-19 Humanitarian platform (www.covid19humanitarian.com) offers free access to all the published field experiences and guidance documents collected. Further datasets generated and analysed may be provided on request.

## Abbreviations

ALNAP: Active Learning Network for Accountability and Performance in Humanitarian Action
CAR: Central African Republic
CDC: Centers for Disease Control and Prevention
CDP: Centre for Disaster Preparedness
DRC: Democratic Republic of the Congo
IASC: Inter Agency Standing Committee
IFRC: International Federation of Red Cross
INGO: International Non-Governmental Organisation
IPC: Infection Prevention and Control
IRC: International Rescue Committee
LRCS: Libyan Red Crescent Society
LSHTM: London School of Hygiene and Tropical Medicine
MHPSS: Mental Health and Psychosocial Support
MoH: Ministry of Health
MSF: Médecins Sans Frontières
NGO: Non-Governmental Organisation
NICE: National Institute for Health and Care Excellence
UN OCHA: United Nations Office for the Coordination of Humanitarian Affairs
PPE: Personal Protective Equipment
RCCE: Risk Communication and Community Engagement
SGBV: Sexual and Gender-Based Violence
UN: United Nation
UNICEF: United Nations Children’s Fund
WASH: Water, Sanitation and Hygiene
WHO: World Health Organization
